# Diet- and Genetically-Induced Obesity Differentially Affect the Fecal Microbiome and Metabolome in Apc^1638N^ Mice

**DOI:** 10.1371/journal.pone.0135758

**Published:** 2015-08-18

**Authors:** Anna C. Pfalzer, Paula-Dene C. Nesbeth, Laurence D. Parnell, Lakshmanan K. Iyer, Zhenhua Liu, Anne V. Kane, C-Y. Oliver Chen, Albert K. Tai, Thomas A. Bowman, Martin S. Obin, Joel B. Mason, Andrew S. Greenberg, Sang-Woon Choi, Jacob Selhub, Ligi Paul, Jimmy W. Crott

**Affiliations:** 1 Cancer Cluster, USDA Human Nutrition Research Center on Aging at Tufts University, Boston, Massachusetts, United States of America; 2 Friedman School of Nutrition Science and Policy, Tufts University, Boston, Massachusetts, United States of America; 3 Agricultural Research Service, USDA Human Nutrition Research Center on Aging at Tufts University, Boston, Massachusetts, United States of America; 4 Neuroscience Department, Tufts University School of Medicine, Boston, Massachusetts, United States of America; 5 Department of Nutrition, University of Massachusetts, Amherst, Massachusetts, United States of America; 6 Phoenix Laboratory, Tufts Medical Center, Boston, Massachusetts, United States of America; 7 Genomics Core, Tufts University School of Medicine, Boston, Massachusetts, United States of America; 8 CHA University School of Medicine, Seoul, South Korea; Charité, Campus Benjamin Franklin, GERMANY

## Abstract

Obesity is a risk factor for colorectal cancer (CRC), and alterations in the colonic microbiome and metabolome may be mechanistically involved in this relationship. The relative contribution of diet and obesity *per se* are unclear. We compared the effect of diet- and genetically-induced obesity on the intestinal microbiome and metabolome in a mouse model of CRC. Apc^1638N^ mice were made obese by either high fat (HF) feeding or the presence of the Lepr^db/db^ (DbDb) mutation. Intestinal tumors were quantified and stool microbiome and metabolome were profiled. Genetic obesity, and to a lesser extent HF feeding, promoted intestinal tumorigenesis. Each induced distinct microbial patterns: taxa enriched in HF were mostly Firmicutes (6 of 8) while those enriched in DbDb were split between Firmicutes (7 of 12) and Proteobacteria (5 of 12). *Parabecteroides distasonis* was lower in tumor-bearing mice and its abundance was inversely associated with colonic Il1b production (p<0.05). HF and genetic obesity altered the abundance of 49 and 40 fecal metabolites respectively, with 5 in common. Of these 5, adenosine was also lower in obese and in tumor-bearing mice (p<0.05) and its concentration was inversely associated with colonic Il1b and Tnf production (p<0.05). HF and genetic obesity differentially alter the intestinal microbiome and metabolome. A depletion of adenosine and *P*.*distasonis* in tumor-bearing mice could play a mechanistic role in tumor formation. Adenosine and *P*. *distasonis* have previously been shown to be anti-inflammatory in the colon and we postulate their reduction could promote tumorigenesis by de-repressing inflammation.

## Introduction

Colorectal cancer (CRC) remains a major public health issue in the US, with approximately 137,000 new cases and 50,000 deaths per year. It is the third most common cancer and third most common cause of cancer deaths [1]. Among the many risk factors for this disease is obesity. Females and males with a BMI of 25–29.9 have relative risks of 1.2 and 1.5 respectively, while those with a BMI of 30 have relative risks of 1.5 and 2.0 [2]. Studies in mice corroborate these epidemiological findings, with both high fat (HF)- [3] and genetically-induced obesity [4] elevating tumor burden. Compelling evidence indicates that elevated colonic inflammation constitutes a major mechanistic link between obesity and CRC [5, 6].

A link between the gut microbiome and colorectal cancer is becoming increasingly apparent and observations that an altered microbiota is already present among individuals with adenomas [7, 8] suggests an involvement at an early stage of carcinogenesis, preceding the appearance of cancer. In one comparison of CRC cases and controls 11 operational taxonomical units (OTUs) belonging to the genera *Enterococcus*, *Escherichia/Shigella*, *Klebsiella*, *Streptococcus and Peptostreptococcus* were significantly more abundant in CRC cases, while 5 OTUs belonging to the genus *Roseburia* and other butyrate-producing bacteria of the family *Lachnospiraceae* were less abundant [9]. Others report a relative enrichment of phylum Bacteroidetes and depletion of Firmicutes was observed in CRC [10].

Studies in which germ-free mice inoculated with stool from tumor-bearing mice had more tumors than those inoculated with stool from tumor-free mice support a causal role for the gut microbiota in colorectal carcinogenesis [11]. Stool from tumor-bearing donor mice was enriched for OTUs of the genera *Bacteroides*, *Odoribacter* and *Turicibacter* as well as a member of the *Erysipelotrichaceae* family and relatively depleted for members of the genus *Prevotella*. Further demonstrating the importance of the colonic microbiota in tumorigenesis, antibiotic administration significantly attenuated tumorigenesis in conventional mice [11]. Interestingly, when repeated using stool from human CRC patients, differences in tumor burden in recipient mice were strongly related to community structure but not to the cancer status of the donor [12]. Genera within the order Bacteroidales (*Parabacteroides* and *Alistipes*), as well as the Verrucomicrobia genus *Akkermansia*, were associated with highest tumor burden while several genera within the order Clostridiales (*Clostridium* Groups XIVa, XI and XVIII, *Flavonifractor*, and unclassified *Lachnospiraceae*) were associated with the lowest tumor burden.

Diet and obesity are potent modulators of the gut microbiome. Mouse studies indicate that both genetically-induced (leptin knockout) [13] and diet-induced obesity promote an expansion of the phylum Firmicutes coincident with a reduction in Bacteroidetes [14]. Human studies comparing lean and obese twins similarly observed a lower abundance of Bacteroidetes but no differences in Firmicutes [15]. Cross-sectional analyses have also linked dietary components with microbial patterns or ‘enterotypes’; an enterotype characterized by high *Bacteroidetes* abundance was highly associated with dietary animal protein, several amino acids and saturated fats while the ‘*Prevotella*’ enterotype was associated with lower intakes for these components but high values for carbohydrates and simple sugars [16].

While these studies have implicated the microbiota as a causal factor in colonic tumorigenesis and have established its sensitivity to diet and obesity, it remains unclear whether dysbiosis explains, at least partly, the elevation in CRC risk associated with obesity. Fully understanding how obesity promotes CRC will require an understanding of not only how obesity affects the composition of the gut microbiota, but also its metabolic capacity and the metabolome of the colonic lumen. To further our understanding of the role of the gut microbiota in obesity-associated CRC and to identify the relative contributions of HF consumption and obesity *per se* to tumorigenesis we compared the effect of HF- and genetic (Lepr^db/db^)-induced obesity on the gut microbiome and intestinal tumorigenesis in Apc^1638N^ mice. Because alterations in the stool metabolome likely play an important role in mediating these phenomena we also profiled the stool metabolome to identify elements that might contribute to the formation of a pro-tumorigeneic milieu in these two modes of obesity.

## Methods

### Animal Study

Animal procedures were approved by the Institutional Animal Care and Use Committee of the Jean Mayer USDA HNRCA at Tufts University. To study tumorigenesis we utilized Apc^1638N^ mice [17] (NCI Mouse Repository. Frederick, MD); heterozygosity for this *Apc* mutation (codon 1638) results in the formation of 1–5 small bowel adenomas or carcinomas by 8 months of age. Although the predilection for developing small, rather than large, intestinal tumors is a common phenomenon in genetically-engineered models of CRC—such as the widely utilized Apc^min^ mouse—the small intestinal tumorigenesis in the Apc^1638N^ animal appears to be highly relevant to colonic carcinogenesis since it responds to dietary modifications like obesity and 1-carbon nutrient depletion in the same fashion as to what occurs in the colon [18, 19].

To study genetically-induced obesity we utilized Lepr^db/db^ mice, which lack a functional leptin receptor and become obese at 3–4 weeks of age [20] (Jackson Laboratory. Bar Harbor, Maine). Wildtype C57BL6/J (Charles River, Wilmington, MA) were also utilized. The following three genotypes were generated: *Apc* ^*+/+*^ *Lepr* ^*+/+*^ (wildtype, Wt), *Apc* ^*+/1638N*^ *Lepr* ^*+/+*^ (Apc) and *Apc* ^*+/1638N*^ *Lepr* ^*db/db*^ (DbDb). Starting at 8 weeks of age, Wt (n = 12) and DbDb (n = 10) mice were fed a low fat (LF) diet while Apc mice were randomized to receive LF (N = 10) or HF (N = 12) diet for 16 weeks. LF and HF diets provided 10 and 60% of calories from fat respectively (Table 1. BioServ, Frenchtown, NJ). Mice were individually housed on a 12 hr light-dark cycle at 23°C and provided *ad libitum* access to water.

**Table 1:** Diet composition.

**Ingredient (g/kg)**	**LF**	**HF**
Casein	210	265
L-Cystine	3	4
Corn Starch	280	0
Maltodextrin	50	160
Sucrose	325	90
Lard	20	310
Soybean Oil	20	30
Cellulose	37.2	65.5
Mineral Mix AIN-93G	35	48
Calcium Phosphate Dibasic	2	3.4
Vitamin Mix AIN-93	15	21
Choline Bitartrate	2.8	3
Total	1000	1000
**Energy (%kcal)**	**LFD**	**HFD**
Carbohydrate	70	21
Protein	20	19
Fat	10	60
Total	100	100

Diets: LF, Low fat. HF, High fat. BioServ (custom) catalog numbers F6654, and F6653 respectively.

Mice were weighed weekly. After 15 weeks on diet, body composition was measured by MRI (EchoMRI, Houston, TX). After 16 weeks on diet, mice were euthanized by CO_2_ asphyxiation followed by cervical dislocation and exsanguination by cardiac puncture. The abdomen was then opened and the small intestine (SI) and large intestines removed onto ice-cold glass plates, opened longitudinally and contents removed. Colon and cecum contents were combined, aliquots frozen in liquid N_2_ and then stored at -80°C. Small and large intestines were then rinsed thoroughly with ice-cold PBS with protease inhibitors (Roche, Indianapolis, IN). The SI and colon were inspected for tumors by a blinded investigator under a dissecting microscope. Tumors were measured before being excised and fixed in formalin for later grading by a rodent pathologist. The remaining normal-appearing SI mucosa was scraped with microscope slides and frozen. Liver, mesenteric fat and gonadal fat depots were also excised, weighed and frozen. Blood was spun at 1000x g and plasma frozen. Plasma insulin and glucose concentrations were measured by ELISA and colorimetric assays respectively (Millipore, Billerica, MA).

To assess colonic inflammation we used a colon organ culture method as previously described [21]. Briefly, two 1 cm sections of the colon were cultured for 24 hr in Dulbecco’s Modified Eagle’s Medium media with protease inhibitors (Roche, Indianapolis, IN) at 37°C with 5% CO_2_. After 24 hr, supernatant was collected and Il1b, Tnf, Il6 and Il4 were measured by electrochemiluminescence array and Sector S600 imager according to manufacturer’s protocols (Mesoscale Discovery, Rockville, MD). Protein concentration was determined by the Bradford assay (Bio-Rad, Hercules, CA).

### Fecal metabolomics

Fecal samples (100 mg) were sent for non-targeted metabolic profiling (Metabolon, Durham, NC) as previously described [22, 23]. Briefly, lyophilized samples were analyzed by three independent platforms; ultrahigh performance liquid chromatography/tandem mass spectrometry (UHPLC/MS/MS) optimized for basic species, UHPLC/MS/MS optimized for acidic species, and gas chromatography/mass spectrometry (GC/MS). Metabolites were identified by automated comparison of the ion features in the experimental samples to a reference library of chemical standard entries that included retention time, mass-to-charge ratio (m/z), preferred adducts, and in-source fragments as well as associated MS spectra, and were curated by visual inspection for quality control using software developed at Metabolon [24]. Missing values were imputed with the compound minimum. Following median scaling and imputation of missing values, statistical analysis of (log-transformed) data was performed.

Metabolomic data were analyzed with MetaboAnalyst 2.0 (http://www.metaboanalyst.ca) [25]. Data were normalized by sum and autoscaled. Heatmap visualization was performed based on Student’s *t*-test results and reorganization of metabolites to show contrast between the groups. Correction for multiple testing was done by calculating false discovery rate (FDR). Principal component analysis (PCA) and partial least-squares discriminant analysis (PLS-DA) were used for classification analyses.

### Fecal microbiome

DNA was extracted from frozen fecal samples using QiaAMP DNA Stool MiniKits (Qiagen, Valencia, CA). The V4 region of the 16S rRNA gene was amplified as previously described [26] and purified using the AMPure XP kit (Agencourt, Indianapolis, IN). Paired-end sequencing (250bp) was performed on an Illumina MiSeq (SanDiego, CA). After quality filtering using Qiime v1.8.0 (http://qiime.org)[27], paired-end sequences were concatenated and demultiplexed. Closed reference OTUs at 99% similarity were assigned using Greengenes [28] and an OTU table generated. The number of sequences were normalized to 41000 (minimum read depth returned) and phylotype-based alpha diversity measures including equitability, number of observed species, Shannon diversity index, Chao-1 and phylogenetic distance were determined. Differences in OTU abundance between groups were identified using LDA (Linear Discriminant Analysis) Effect Size (Lefse) and Multivariate Association with Linear Models (MaAsLin) tools [29](http://huttenhower.sph.harvard.edu/galaxy/).

### Gene expression

We profiled the expression of genes encoding adenosine-metabolizing enzymes in the small intestinal mucosa: adenosine deaminase (*Ada*), adenosine kinase (*Adk*), ectonucleoside triphosphate diphosphohydrolases (*Entpd1*, *Entpd3*, *Entpd8*), purine nucleoside phosphoylases (*Pnp*, *Pnp2*), S-adenosylhomocysteine hydrolase (*Ahcy*), deoxycytidine kinase (*Dck*) and 5’ nucleotidases (*Nt5c*, *Nt5c1a*, *Nt5c1b*, *Nt5c2*, *Nt5c3*, *Nt5c3b*, *Nt5e*, *Nt5m*). Total RNA was isolated from small intestinal scrapings using Trizol reagent and cDNA synthesized using Superscript III reverse transcriptase. Real-time PCR was performed using SYBR green master mix (Life technologies, Grand Island, NY) and an ABI7300 thermocyler (Applied Biosystems, Foster City, CA). Primer sequences were obtained from qPrimerDepot (http://mouseprimerdepot.nci.nih.gov/) or NCBI Primer Blast [30] (S1 Table). Relative expression was calculated using the 2^-ΔΔCt^ method and statistical analyses were performed on ΔCt values. *Gapdh* was used as the control gene.

### Statistics

All data are reported as mean ± SEM. Statistical calculations were performed in Systat (San Jose, CA) and R [31]. Between groups comparisons were made with ANOVA, 2way ANOVA or t-test where appropriate. Associations between variables were assessed by linear regression. Significance was accepted when p<0.05 or, when multiple comparisons conducted, a False Discovery Rate cutoff of q< 0.2 or less was used. Cluster analysis and heatmaps were generated with CIMminer [32].

## Results

### Physiology

**Fig 1:**
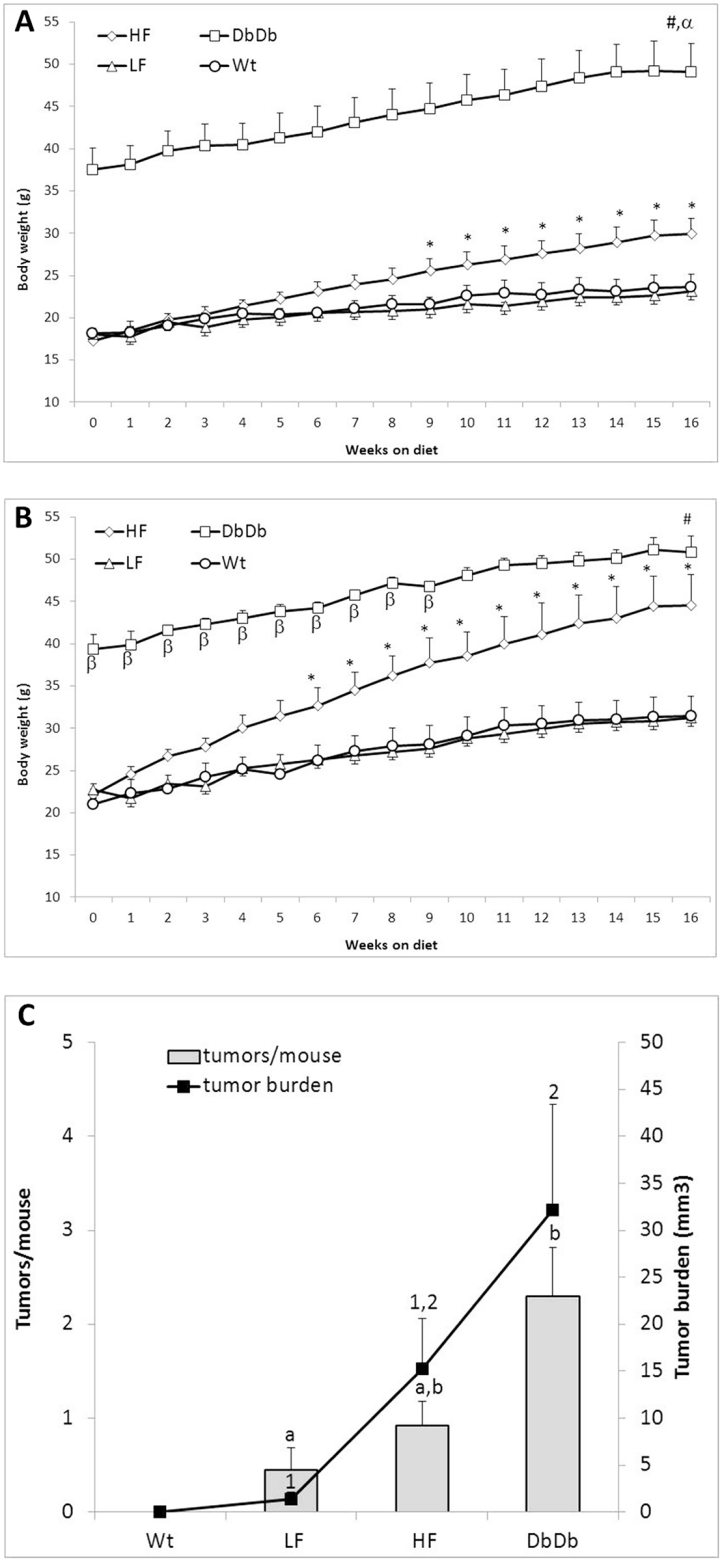
Effect of diet and genotype on body weight and tumor burden. A) Weight of *female* mice by group. * p<0.05 vs. LF, # p<0.05 vs. LF (all time points), α p<0.05 vs. HF (all time points). B) Weight of *male* mice by group. * p<0.05 vs. LF, # P<0.05 vs. LF (all time points), β p<0.05 vs. HF. C) Small intestinal tumor burden by group. p_trend_ <0.001 for tumor number and burden. Groups with different number are significantly different by post-test (p<0.05).

HF consumption significantly increased body weight (Fig 1A and 1B). Although the dietary interventions were begun when all animals were at the same age, DbDb mice initially were approximately double the body weight of all other mice and continued to gain weight thereafter. At week 15 fat mass was significantly higher in DbDb mice than LF mice. Although numerically higher in females, HF feeding significantly elevated fat mass only in male mice. Liver weight, insulin and glucose were significantly elevated in DbDb mice but not in HF fed mice. Lean mass was not altered by HF fed or DbDb mice (Table 2).

**Table 2:** Physiological characteristics of mice by group.

Endpoint	Wt		LF		HF		DbDb		2Way ANOVA P	
	M (7)	F (5)	M (4)	F (5)	M (4)	F (8)	M (3)	F (7)	Group	Sex
Body weight (g)	31.31±2.33	23.58±1.42	30.83±1.80	22.60±0.46	44.41±3.66*	29.73±1.83	51.13±1.38*	49.17±3.51*	<0.0001	<0.0001
Total fat mass (g)	8.05±1.70	5.07±0.89	7.60±1.41	4.85±1.04	19.13±3.08*	9.94±1.96	28.22±1.04*	26.51±1.93*	<0.0001	0.006
Total lean mass (g)	18.95±0.85	15.24±0.86	18.66±0.32	14.27±1.14	21.06±1.17	16.75±0.37	18.89±0.62	17.39±1.56	0.1	<0.0001
Mesenteric fat (g)	0.55±0.11	0.28±0.07	0.41±0.07	0.28±0.04	1.23±0.28*	0.39±0.09	1.04±0.16	1.00±0.13*	<0.0001	0.002
Gonadal fat (g)	1.01±0.24	0.58±0.15	0.97±0.18	0.52±0.09	2.48±0.26*	1.53±0.35	1.57±0.18	1.84±0.26*	<0.0001	0.063
Liver (g)	1.29±0.17	0.94±0.10	1.19±0.06	1.04±0.07	1.33±0.15	0.96±0.04	4.93±0.30*	3.63±0.29*	<0.0001	<0.0001
Plasma insulin (ng/ml)	3.10±1.26	0.94±0.16	1.70±0.23	1.20±0.20	4.09±1.91	1.09±0.18	11.44±0.82	16.03±2.43*	<0.0001	0.8
Plasma glucose (μM)	8.09±0.87	5.08±2.56	8.21±0.42	7.78±0.47	11.19±1.07	10.05±0.92	20.81±1.73*	18.26±2.51*	<0.0001	0.1

Total lean and fat mass measured by MRI. M, male; F, female. Samples size in parentheses.

* p<0.05 vs LF (of same sex).

### Intestinal Tumors

No tumors were observed in the SI of Wt mice. Amongst Apc mice, the SI tumor incidence was 33%, 67% and 100% in LF, HF and DbDb mice respectively (x^2^ P = 0.008). A similarly significant step-wise increase in tumor multiplicity and burden was also observed (Fig 1C). Sex-specific data are reported in S2 Table. No tumors were observed in the colon of any mouse. All tumors were confirmed to be adenomatous polyps.

### Fecal Microbiome

Population diversity was assessed via several metrics. Significant between-group differences were found with Observed Species and PD whole tree metrics (p<0.05), and a trend was apparent for Chao index (p = 0.064). For these analyses the HF group had the lowest numerical value of population diversity, which attained significance in comparison with the DbDb group. No significant differences were observed between groups for Shannon index or Equitability index (p> 0.05). When comparing between groups at a phylum level there were no significant differences in the four major phyla present (Actinobacteria, Bacteroidetes, Firmicutes, Proteobacteria) or in the ratio of Firmicutes to Bacteroidetes (ANOVA p>0.05).

**Fig 2:**
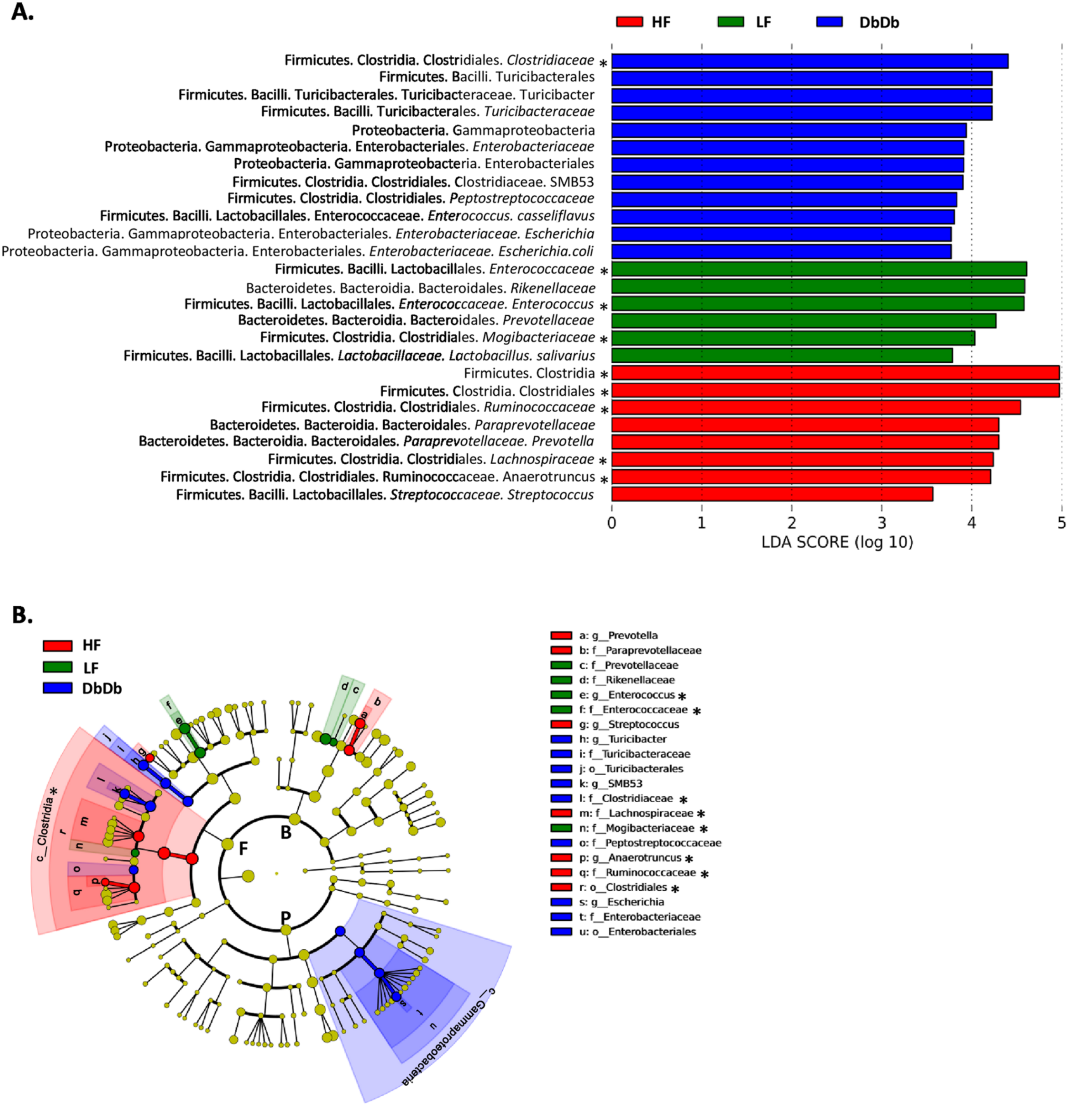
LDA effect size analysis of between group differences in stool bacterial abundances in Apc^1638N^ mice. A) Output showing effect size of 29 significantly enriched taxa in each group. Model = group x gender. B) Significant taxa plotted onto a cladogram. F, Firmicutes; P, Proteobacteria; B, Bacteroidetes; c_, class; o_, order; f_, family; g_genus, s_, species. * taxa that were also associated with that group in the MaASLin analysis. N = 29.

LEfSe analysis was performed on data from Apcmice, identifying 26 significantly enriched taxa across three phyla; 6, 8 and 12 taxa enriched in LF, HF and DbDb mice respectively (Fig 2A and 2B). Firmicutes featured prominently amongst those enriched in both modes of obesity (6 of 8 and 7 of 12 for HF and DbDb respectively). For HF mice, the remainder of the defining taxa were Bacteroidetes (2 of 8), while for DbDb the remainder were Proteobacteria (5 of 12). MaAsLin analysis facilitated the parsing out of associations with genotype, diet, sex and tumor number (Table 3). In agreement with the LEfSe analysis, the family *Clostridiacea* (phyla Firmicutes) was associated with the DbDb genotype; families *Ruminococcaceae* and *Lachnospiracea* (both phyla Firmicutes) were associated with HF diet and the family *Enterococcaceae* (phyla Firmicutes) was associated with the LF diet. In addition several OTUs from the phyla Firmicutes and Bacteroidetes were associated with each sex.

**Table 3:** Multivariate Association with Linear Models (MaAsLin) output.

Variable	Feature (OTU)	Coefficient	P-value	Q-value
Apc WT	p__ Actinobacteria|c__Actinobacteria	0.00	0.004	0.097
Apc WT	p__Proteobacteria|c__Gammaproteobacteria|o__Pseudomonadales	0.00	0.010	0.163
Apc WT	p__Bacteroidetes|c__Bacteroidia|o__Bacteroidales|f__Paraprevotellaceae	-0.08	0.012	0.163
Apc WT	p__Bacteroidetes|c__Bacteroidia|o__Bacteroidales|f__Paraprevotellaceae|g__Prevotella	-0.08	0.012	0.163
Apc WT	p__Actinoc__Actinoo__Bifidobacteriales|f__Bifidobacteriaceae	0.00	0.013	0.163
Apc WT	p__Actinoc__Actinoo__Bifidobacteriales|f__Bifidobacteriaceae|g__Bifidobacterium	0.00	0.013	0.163
Apc WT	p__Firmicutes|c__Clostridia|o__Clostridiales|f__Peptococcaceae	-0.02	0.018	0.197
DbDb WT	p__Firmicutes|c__Clostridia|o__Clostridiales|f__Clostridiaceae| g__Sarcina	0.00	0.002	0.055
DbDb WT	p__Bacteroidetes|c__Bacteroidia|o__Bacteroidales|f__Paraprevotellaceae	0.09	0.007	0.128
DbDb WT	p__Bacteroidetes|c__Bacteroidia|o__Bacteroidales|f__Paraprevotellaceae|g__Prevotella	0.09	0.007	0.128
DbDb WT	p__Bacteroidetes|c__Bacteroidia|o__Bacteroidales|f__Rikenellaceae	0.12	0.014	0.170
DbDb WT	p__Bacteroidetes|c__Bacteroidia|o__Bacteroidales|f__Prevotellaceae	0.01	0.014	0.170
DbDb WT	**p__Firmicutes|c__Clostridia|o__Clostridiales|f__Clostridiaceae**	-0.10	0.017	0.192
DbDb WT	p__Firmicutes|c__Bacilli|o__Lactobacillales|f__Carnobacteriaceae	0.00	0.018	0.197
LF Diet	**p__Firmicutes|c__Clostridia|o__Clostridiales|f__Ruminococcaceae**	-0.11	4.03E-05	0.024
LF Diet	**p__Firmicutes|c__Clostridia|o__Clostridiales|f__Ruminococcaceae|g__Anaerotruncus**	-0.01	0.000	0.040
LF Diet	**p__Firmicutes|c__Bacilli|o__Lactobacillales|f__Enterococcaceae|g__Enterococcus**	0.17	0.000	0.040
LF Diet	**p__Firmicutes|c__Bacilli|o__Lactobacillales|f__Enterococcaceae**	0.17	0.001	0.040
LF Diet	p__Firmicutes|c__Bacilli|o__Lactobacillales|f__Enterococcaceae|g__Enterococcus|s__casseliflavus	0.01	0.001	0.040
LF Diet	p__Firmicutes|c__Clostridia|o__Clostridiales|f__Lachnospiraceae|g__Roseburia	-0.03	0.001	0.040
LF Diet	p__Firmicutes|c__Clostridia|o__Clostridiales|f__Peptostreptococcaceae	0.03	0.001	0.040
LF Diet	**p__Firmicutes|c__Clostridia|o__Clostridiales|f__Mogibacteriaceae**	0.01	0.002	0.062
LF Diet	p__Bacteroidetes|c__Bacteroidia|o__Bacteroidales|f__S24-7	0.11	0.005	0.114
LF Diet	p__Firmicutes|c__Bacilli|o__Turicibacterales	0.05	0.011	0.163
LF Diet	p__Firmicutes|c__Bacilli|o__Turicibacterales|f__Turicibacteraceae	0.05	0.011	0.163
LF Diet	p__Firmicutes|c__Bacilli|o__Turicibacterales|f__Turicibacteraceae| g__Turicibacter	0.05	0.011	0.163
LF Diet	p__Firmicutes|c__Clostridia|o__Clostridiales|f__Clostridiaceae|g__SMB53	0.06	0.012	0.163
LF Diet	**p__Firmicutes|c__Clostridia|o__Clostridiales|f__Lachnospiraceae**	-0.06	0.012	0.163
LF Diet	p__Firmicutes|c__Clostridia|o__Clostridiales|f__Lachnospiraceae| g__Coprococcus	-0.02	0.012	0.163
LF Diet	p__Firmicutes|c__Bacilli	0.17	0.014	0.170
LF Diet	**p__Firmicutes|c__Clostridia**	-0.15	0.017	0.192
LF Diet	**p__Firmicutes|c__Clostridia|o__Clostridiales**	-0.15	0.017	0.192
Male Sex	p__Firmicutes	0.15	0.000	0.040
Male Sex	p__Firmicutes|c__Clostridia|o__Clostridiales|f__Lachnospiraceae|g__Dorea	-0.01	0.001	0.040
Male Sex	p__Bacteroidetes|c__Bacteroidia|o__Bacteroidales|f__Porphyromonadaceae	-0.08	0.001	0.040
Male Sex	p__Bacteroidetes|c__Bacteroidia|o__Bacteroidales|f__Porphyromonadaceae|g__Parabacteroides	-0.08	0.001	0.040
Male Sex	p__Bacteroidetes	-0.16	0.001	0.040
Male Sex	p__Bacteroidetes|c__Bacteroidia	-0.16	0.001	0.040
Male Sex	p__Bacteroidetes|c__Bacteroidia|o__Bacteroidales	-0.16	0.001	0.040
Male Sex	p__Bacteroidetes|c__Bacteroidia|o__Bacteroidales| f__Porphyromonadaceae|g__Parabacteroides|s__distasonis	-0.07	0.003	0.083
Male Sex	p__Firmicutes|c__Clostridia|o__Clostridiales|f__Dehalobacteriaceae	-0.01	0.008	0.150
Male Sex	p__Firmicutes|c__Clostridia|o__Clostridiales|f__Dehalobacteriaceae|g__Dehalobacterium	-0.01	0.009	0.163
Male Sex	p__Firmicutes|c__Clostridia|o__Clostridiales|f__Lachnospiraceae|g__Coprococcus	-0.02	0.013	0.163
Male Sex	p__Firmicutes|c__Bacilli	0.14	0.014	0.170
Tumor #	p__Bacteroidetes|c__Bacteroidia|o__Bacteroidales|f__Porphyromonadaceae	-0.05	0.001	0.040
Tumor #	p__Bacteroidetes|c__Bacteroidia|o__Bacteroidales|f__Porphyromonadaceae|g__Parabacteroides	-0.05	0.001	0.040
Tumor #	**p__Bacteroidetes|c__Bacteroidia|o__Bacteroidales|f__Porphyromonadaceae|g__Parabacteroides|s__distasonis**	-0.04	0.001	0.051
Tumor #	p__Actinobacteria|c__Actinobacteria|o__Actinomycetales|f__Corynebacteriaceae	0.02	0.004	0.093
Tumor #	p__Actinobacteria|c__Actinobacteria|o__Actinomycetales|f__Corynebacteriaceae|g__Corynebacterium	0.02	0.004	0.093
Tumor #	p__Bacteroidetes	-0.09	0.004	0.093
Tumor #	p__Bacteroidetes|c__Bacteroidia	-0.09	0.004	0.093
Tumor #	p__Bacteroidetes|c__Bacteroidia|o__Bacteroidales	-0.09	0.004	0.093
Tumor #	p__Actinobacteria|c__Actinobacteria|o__Actinomycetales|f__Micrococcaceae|g__Arthrobacter	0.01	0.004	0.097
Tumor #	p__Actinobacteria|c__Actinobacteria|o__Actinomycetales	0.02	0.005	0.105
Tumor #	p__Firmicutes|c__Bacilli|o__Lactobacillales|f__Aerococcaceae|g__Aerococcus	0.02	0.010	0.163
Tumor #	p__Firmicutes	0.06	0.012	0.163

Model = Apc (Mut or Wt) x DbDb (Mut or Wt) x Diet (LF or HF) x Sex (M or F) x Tumors (number of tumors present). Abbreviations: Mut, mutant; Wt, wildtype; LF, low fat; HF, high fat; p_, phylum; c_, class; o_, order; f_, family; g_, genus; s_, species. N = 41 (includes WtWt mice). Taxa in bold were also identified to be associated with that trait (variable) in the LDA effect size analysis.

MaAsLin analysis also identified OTUs both positively (phyla Firmicutes and Actinobacteria) and negatively (phyla Bacteroidetes) associated with tumor number. Amongst these, *Parabacteroides distasonis* was also identified by LefSe analysis as being lower in tumor-bearing mice. Further, t-test (p = 0.02) and regression analyses (R = -0.31, p = 0.04) corroborate a depletion of *P*.*distasonis* in tumor-bearing mice and with increasing tumor number respectively. *P*.*distasonis* abundance was also inversely related to colonic production of Il1b (R = -0.34, p = 0.05) but not Tnf, Il6 or Il4 (p>0.05) (S1 Fig).

### Fecal metabolome

**Fig 3:**
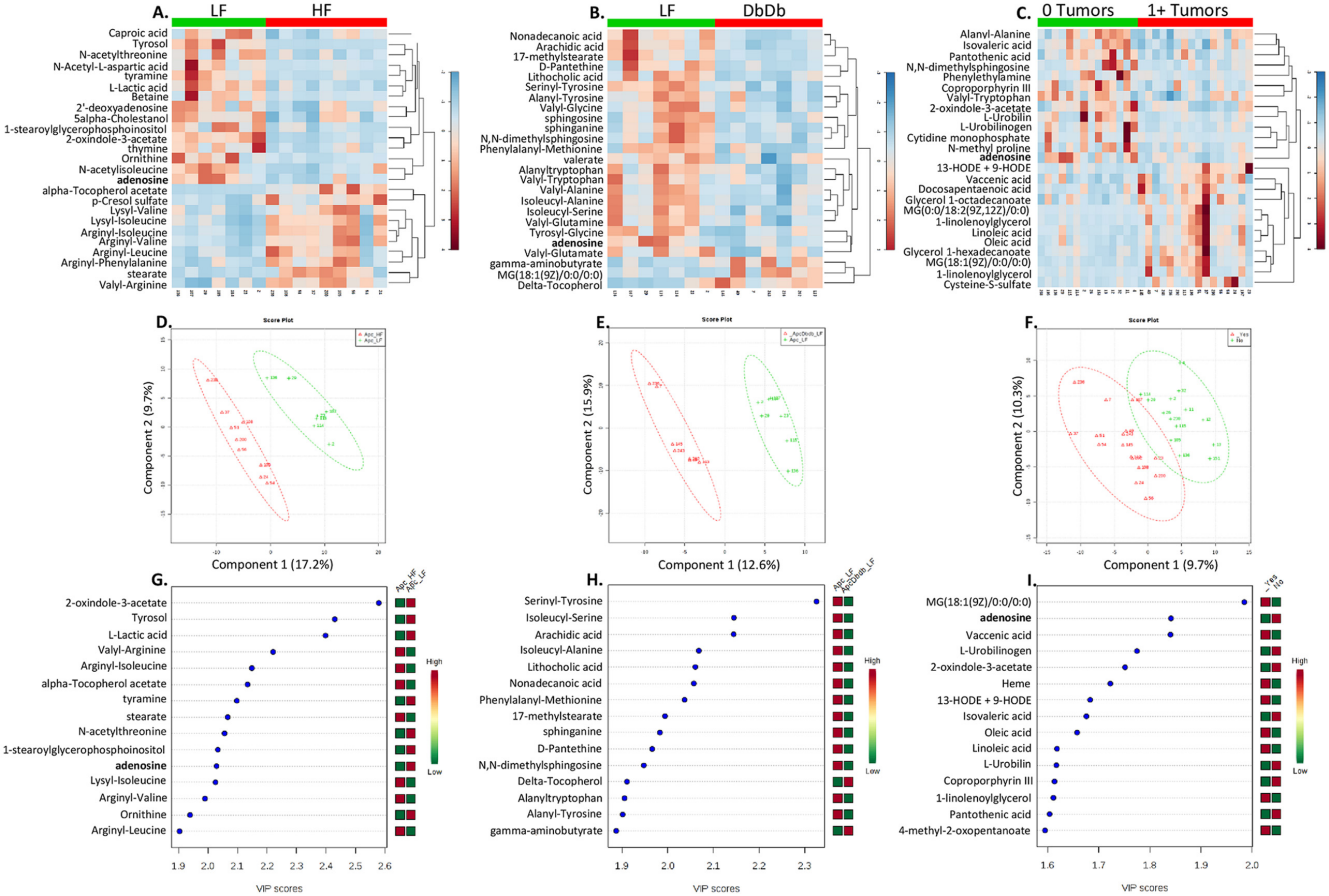
Impact of obesity and intestinal tumor presence on the fecal metabolome of mice. Low and high fat fed mice are compared in the first column (A,D,G). Low fat fed and genetically obese mice are compared in the second column (B,E,H). Mice with and without intestinal tumors are compared in the third column (C,F,I). The top row (A-C) shows heat maps of top 25 most significantly different metabolites for each comparison (p<0.05); color represents normalized metabolite concentration from low (blue) to high (red). The second row (D-F) shows discrimination of groups using partial least squares discriminate analysis. The third row (G-I) shows the metabolites most strongly influencing discrimination by the partial least squares discriminate analysis. The Variable Importance In Projection (VIP) score is the weighted sum of squares for the partial least-squares loadings with the amount of variance explained by each component taken into account.

415 metabolites were detected in fecal samples. Comparing (Apc) LF and HF fed Apc mice, 49 metabolites returned a p value of <0.05 and 14 with a q<0.2 (Fig 3A). Comparing LF and DbDb Apc mice 41 metabolites returned a p value of <0.05 but none attained a q<0.2 (Fig 3B). Using the relaxed cut-off of p<0.05, 5 metabolites were changed in both comparisons: adenosine, 2-oxindole-3-acetate, caproic acid, arachadic acid and tyrosyl glycine. Comparing mice with and without tumors, 29 metabolites returned a p value of <0.05 but none attained a q<0.2 (Fig 3C). Adenosine and 2-oxindole-3-acetate were altered in all three comparisons.

Because previous studies have demonstrated an anti-inflammatory role for adenosine in the colon we tested its association with inflammatory cytokines in the colon. Consistent with this role, fecal adenosine concentrations were inversely associated with the production of Tnf (R = -0.5, p = 0.01) and Il1b (R = -0.73, p = 1.3x10^-5^) but not Il4 or Il6 (p>0.05) (S1 Fig).

Adenosine participates in endogenous reactions that form AMP, adenine, inosine and S-Adenosyl homocysteine (SAM). To identify possible mechanisms for the observed depletion of adenosine, we assessed whether its concentration was related to that of any immediately related metabolites or to the expression of genes encoding adenosine-metabolizing enzymes. Although AMP and SAM were not detected in our samples, fecal adenosine levels were positively associated with inosine (R = 0.5, p = 0.009) but not adenine (R = 0.05, p = 0.8) concentrations. Interestingly, inosine and hypoxanthine were inversely related (R = -0.53, p = 0.006), while hypoxanthine and adenine concentrations were positively related (R = 0.44, p = 0.03). Fecal adenosine concentration was not significantly associated with the expression of any adenosine-metabolizing gene in the small intestinal mucosa (p>0.05).

Using the relaxed cut-off for metabolites, PLS-DA could effectively separate HF and DbDb groups from the LF group (Fig 3D and 3E). The metabolites that most heavily drove the separation were 2-oxindole-3-acetate, tyrosol and lactic acid for the HF vs. LF comparison and serinyl tyrosine, isoleucyl serine and arachidic acid for the DbDb vs LF comparison (Fig 3G and 3H). Similarly mice with and without tumors could be distinguished in this analysis, with oleic acid, adenosine and vaccenic acid being most influential (Fig 3F and 3I). In contrast, PCA could not effectively distinguish groups in these two comparisons (data not shown).

### Integrative analysis

Correlation analysis between all OTUs and metabolites identified 107 metabolites and 31 OTUs had at least one significant association (q<0.05). We performed a cluster analysis of the correlation R values and observed two clear clusters of bacteria, suggesting similarities in their metabolic capacities and/or requirements with regard to this subset of detectable metabolites (S2 Fig). Cluster 1 was comprised mostly of members of the Firmicutes class Bacilli while Cluster 2 is made up of 3 classes of Proteobacteria (beta, delta and gamma), Firmicutes class Clostridia and Phyla TM7class TM7-3.

Although the concentration of adenosine was not significantly associated with the abundance of any OTU (q>0.2), its immediate precursor adenine was strongly associated with the genus *Lactobacillus* (R = 0.75, q = 0.002) and 3 other higher order taxa associated with this genus (family *Lactobacillaceae*, order Lactobacillales and Class bacilli. R = 0.75–0.65, q = 0.002–0.03).

## Discussion

In the current study we show that obesity that is driven by either HF feeding or by genetic mutation promotes intestinal tumorigenesis. Although prior studies have demonstrated the pro-tumorigenic effects of these two means of producing obesity [3, 4], the two modalities have not previously been compared side-by-side under parallel experimental conditions. Although the high dietary fat content in the HF group might, by itself, have driven tumorigenesis since dietary fat activates pro-inflammatory TLR receptors in the colon [33], our observations underscore the fact that even in the absence of excess dietary fat, obesity *per se* enhances tumorigenesis.

At a phylum level we did not observe any between-group differences in the four major phyla present: Firmicutes, Bacteroidetes, Proteobacteria and Actinobacteria. Similarly, no phylum level differences were observed between mice with and without tumors. Unlike others [13, 14], we did not observe an alteration in the ratio of Firmicutes: Bacteroidetes in either type of obesity: this absence in a shift in the Firmicutes: Bacteroidetes ratio agrees with recent studies of human stool, which also failed to detect such differences [34]. LDA effect size analysis identified 26 significantly discriminative features across three phyla. Interestingly, although we didn’t detect differences in the total abundance of each phyla, species enriched in HF mice were mostly Firmicutes (class clostridia) while those enriched in DbDb mice were split between Firmicutes and Proteobacteria (class Gamma Proteobacteria)(Fig 2B). In contrast to recent studies showing a depletion of *Ruminococcaceae* in HF fed male mice [35], we observed an enrichment of this family in our study (Fig 2A).

A potentially important observation of ours was the significant depletion of the species *Parabacteroides distasonis* in those mice who harbored tumors. Although there are no reports linking this species to CRC, there is evidence that *P*.*distasonis* has anti-inflammatory effects in the colon. In patients with Crohn’s disease, *P*.*distasonis* is more frequently absent than in those who are disease free, and, in those with Crohn’s, it is more frequently absent in those with severe compared to mild inflammation [36]. A *bona fide* anti-inflammatory role for this species is supported by data showing that oral administration of a membrane fraction of *P*.*distasonis* significantly attenuated dextran sodium sulfate-induced colitis in mice [37]. Moreover, these authors demonstrated that the membrane fraction of *P*.*distasonis* reduced the release of Tnf, Il6, Ccl2 (MCP-1) and Ccl12 (MCP-5) by RAW264.7 macrophages after LPS challenge. Consistent with this anti-inflammatory role, the relative abundance of *P*.*distasonis* was inversely associated with colonic production of the inflammatory cytokine Il1b (R = -0.34, p = 0.05) in our own study. One of the prevailing theories as to how obesity promotes colorectal carcinogenesis is by producing a chronic, low-grade state of inflammation in the colon [5, 6]; a reduced abundance of this organism mice may therefore play a mechanistic role in enhancing tumorigenesis. Because the anti-inflammatory properties of *P*.*distasonis* were demonstrated with a membrane fraction [37] and its abundance was not significantly related to any metabolites in the current study, we suggest that its putative protective effect is more likely related to an immune-modulatory capacity of specific membrane components rather than the production of chemoprotective metabolites.

While both modes of obesity appeared to cause a similar degree of impact, in terms of taxa with altered abundance, HF feeding was more perturbing to the metabolome than genetic obesity (Fig 3). For some of the differentially abundant metabolites it is likely that differences in stool may be directly related to amounts of those nutrients present in the diet consumed. For example, the higher abundance of alpha-tocopherol may be explained by a higher relative amount of vitamin mix added to the HF diet. However, other metabolite changes could result from diet-microbial interactions. For example, the HF fed mice have relatively less lactate (q = 0.03) and specific taxa belonging to the order Lactobacillales (Table 3), which as their name suggests, produce lactate from sugars. Their altered abundance, in turn, may be related to the fact that the HF diet has substantially less sucrose than the LF diet (325 vs. 90 g/kg). To assess microbiome-metabolite interactions systematically, we correlated all metabolites against all OTUs and found that 107 metabolites correlated with at least one OTU and 31 OTUs correlated with at least one metabolite (S2 Fig). This analysis clearly identified two major clusters of taxa with apparently opposite substrate requirements or metabolite production. Interestingly, these clusters segregated based on bacterial class; the first cluster is mostly Bacilli (phylum Firmicutes) while the second was a combination of alpha, beta and gamma proteobacteria (phylum Proteobacteria), clostridia (phylum Firmicutes) and TM7-3 (phylum TM7).

When considering all three comparisons i.e. LF vs. HF, LF vs. DbDb and Tumor No vs. Yes, only two metabolites were found to be altered in all three; 2-oxindole-3 acetate and adenosine. Very little information is available on the former; however there is an abundance of literature demonstrating an anti-inflammatory role of adenosine in the colon. Specifically, interventions to increase adenosine signaling, either by restoring endogenous adenosine by inhibiting its breakdown [38–40], or by agonizing adenosine receptors [41–43] have been shown to attenuate inflammation in rodent colitis models. Conversely, decreasing adenosine signaling by reducing endogenous adenosine [44] or knocking out [45] or antagonizing [45] adenosine receptors increases inflammation in these models. Consistent with this anti-inflammatory role, in our study fecal adenosine was inversely associated with mucosal production of pro-inflammatory cytokines Tnf (R = -0.5, p = 0.01) and Il1b (R = -0.73, p = 1.3x10^-5^).

There is a large body of evidence that existing cancers use adenosine to suppress anti-tumor immunity (reviewed in [46]). Specifically, tumors recruit and activate T_reg_, myeloid-derived suppressor cells and M2 macrophages which express CD39 and/or CD73 [47]. These ectonucleotidases convert AMP to adenosine on the cell surface which subsequently suppresses natural killer cells and effector T cells via the A2A receptor [48], thus suppressing the anti-tumor response. Adenosine also stimulates VEGF production by M2 macrophages, promoting angiogenesis [47]. Based on our data and the substantial body of evidence that adenosine is anti-inflammatory in the colon, we suggest that early in tumorigenesis this same immune suppression activity of adenosine may, paradoxically, have an anti-tumor effect by suppressing inflammation-induced initiation. Extrapolating from this, we suggest that a depletion of adenosine, such as that observed in our obese mice, may contribute to the formation of a pro-carcinogenic milieu by de-repressing inflammation.

In order to gain an understanding of the mechanism for the observed depletion of adenosine depletion we measured the expression of all endogenous genes encoding adenosine-metabolizing enzymes in the small intestinal mucosa. Others have previously reported that genetic-induced obesity reduces the expression of *Cd39* and *Cd73* and diet-induced obesity reduced *Cd73* in epididymal fat of mice [49]. These ectonucleotidases cleave ATP to adenosine and a reduced intestinal expression of these could account for the reduced fecal adenosine concentration observed. We found no association between intestinal *Cd39* or *Cd73* expression and adenosine concentrations, neither were any other adenosine-metabolizing genes related to its concentration. One possible metabolic fate for adenosine is its sequential metabolism to inosine, hypoxanthine and adenine. We noted a robust positive association of adenosine with inosine, negative association of inosine with hypoxanthine and positive association of hypoxanthine with adenine. Thus alterations in adenosine concentration could be due to flux through this pathway. While fecal adenosine was not correlated with the abundance of any bacterial taxa (q>0.2), adenine concentrations were positively related to the class Bacilli, order Lactobacillales, family *Lactobacillaceae* and genus *Lactobacillus* (S2 Fig q<0.05 R = 0.65–0.75). Thus it is conceivable that fluctuations in the abundance of these taxa could alter adenosine concentrations through hypoxanthine and inosine.

Weir et al profiled the fecal metabolome of human subjects with and without CRC and identified 22 biochemicals with differing abundances [50]. Interestingly, there is little overlap in these changes with those we observed. Further, for three metabolites changes occurred in the opposite direction between the two studies (oleic acid, linoleic acid and monooleoglycerol). Such discrepancies are not surprising in a human versus mouse comparison and since dietary differences were equally distinct. One limitation of the current study is that tumors formed in the small intestine, while microbes and metabolites were profiled downstream in the cecum and colon. Thus, while some of the metabolites and taxa detected may have been present at the site of tumor formation, others may have been unique to the colon and cecum and as such are unlikely directly to affect tumorigenesis.

In summary, our studies have confirmed the tumor promoting effect of diet and genetic-induced obesity in a mouse model of CRC. Profiling the gut microbiome revealed distinct patterns for each model of obesity implying that the microbiome is sensitive to both diet and host physiology. The lack of consistency between studies profiling the effect of obesity on the microbiome highlights the high model and environment specificity. Nevertheless, in our model we observed a depletion of the species *P*.*distasonis* in tumor-bearing mice, and we postulate that this may have important functional implications given its known anti-inflammatory actions. We noted an inverse association between the abundance of this species and colonic Il1b production, which is consistent with data from others demonstrating anti-inflammatory effects of this species in the colon. Metabolomic profiling also revealed relatively distinct patterns in response to these different models of obesity. Several metabolites were changed in both models and, amongst these; the nucleoside adenosine was also lower in tumor-bearing mice. Considerable data has established the anti-inflammatory role of adenosine in the colon, and its inverse association with colonic Il1b and Tnf production in our study is consistent with this. Based on these data we suggest that a depletion of adenosine and *P*.*distasonis* could be permissive to the development of intestinal inflammation, thereby promoting tumorigenesis. Further work is required to confirm this supposition, but if true, strategies to restore, replace or agonize the same signaling pathways could possess some utility in reducing colonic inflammation and thereby reducing the risk for CRC in those with elevated levels of inflammation including the obese.

## Supporting Information

S1 Fig Association of fecal adenosine concentration and Parabacteroides distasonis abundance with inflammatory cytokine production by the colonic mucosa. Normalized adenosine concentration in fecal matter correlates with Il1b (A) and Tnf (B) but not Il4 (C) and Il6 (D) production in *ex vivo* colonic tissue. Relative abundance of *Parabacteroides distasonis* in fecal matter correlates with Il1b (E) but not Tnf (F), Il4 (G) and Il6 (H) production in *ex vivo* colonic tissue.
(DOCX)

S2 Fig Heat map of microbiome–metabolome interactions. Heat map consist of 107 metabolites significantly correlated with at least one OTU and 31 OTUs significantly correlated with at least 1 metabolite (q< 0.05). Color intensity indicates R value of association. For brevity phylum, class and lowest taxonomic classification of each OUT is listed (ie order, family and genus are omitted if OTU is a species). Metabolites by row: 1, 4-hydroxyphenylacetate; 2, betaine; 3, tyramine; 4, tyrosol; 5, lactate; 6, alpha-hydroxyisocaproate; 7, 2-hydroxy-3-methylvalerate; 8, N-acetylaspartate (NAA); 9, **adenine**; 10, N-acetylhistidine; 11, guanosine; 12, alpha-CEHC glucuronide; 13, 2-hydroxybutyrate (AHB); 14, lysylvaline; 15, 4-hydroxyphenylpyruvate; 16, lysylisoleucine; 17, glycylvaline; 18, tryptophylisoleucine; 19, serylphenyalanine; 20, alanylleucine; 21, alanylphenylalanine; 22, isoleucylleucine; 23, valylleucine; 24, valylphenylalanine; 25, threonylphenylalanine; 26, serylleucine; 27, isoleucylphenylalanine; 28, valyltyrosine; 29, tyrosylleucine; 30, asparagylleucine; 31, histidylleucine; 32, phenylalanylleucine; 33, isoleucyltyrosine; 34, histidyltyrosine; 35, tryptophylleucine; 36,seryltyrosine; 37, leucylleucine; 38, leucylphenylalanine; 39, val-val-val; 40,phenylalanylalanine; 41, leucylisoleucine; 42, phenylalanylvaline; 43, leucylthreonine; 44, leucylalanine; 45, valylisoleucine; 46, tyrosylvaline; 47, leucylmethionine; 48, methionylvaline; 49, threonylisoleucine; 50, leucylserine; 51, threonylvaline; 52, isoleucylisoleucine; 53, isoleucylvaline; 54, asparagylisoleucine; 55, phenylalanyltyrosine; 56, histidylisoleucine; 57, phenylalanylisoleucine; 58, tyrosylisoleucine; 59, leucylglycine; 60, valylvaline; 61, erucate (22:1n9); 62, 15-methylpalmitate (isobar with 2-methylpalmitate); 63, 2-oleoylglycerol (2-monoolein); 64, 1-stearoylglycerol (1-monostearin); 65, docosapentaenoate (n3 DPA 22:5n3); 66, alanine; 67, glucosylglycerol; 68, 3-hydroxypropanoate; 69, 1-palmitoylglycerophosphoglycerol; 70, uracil; 71, beta-alanine; 72, hypoxanthine; 73, phenylacetate; 74, adrenate (22:4n6); 75, palmitoleate (16:1n7); 76, 2-myristoylglycerol (2-monomyristin); 77, conjugated linoleate (18:2n7 9Z11E); 78, docosadienoate (22:2n6); 79, cis-vaccenate (18:1n7); 80, glycerol; 81, oleate (18:1n9); 82, linoleate (18:2n6); 83, palmitate (16:0); 84, linolenate [alpha or gamma (18:3n3 or 6)]; 85 1-linolenoylglycerol; 86, 2-linoleoylglycerol (2-monolinolein); 87, 1-palmitoylglycerol (1-monopalmitin); 88, 1-oleoylglycerol (1-monoolein); 89,2-palmitoylglycerol (2-monopalmitin); 90, 1-arachidonylglycerol; 91, 10-heptadecenoate (17:1n7); 92, 1-stearoylglycerophosphoserine; 93, 1-stearoylglycerophosphoethanolamine; 94, 1-palmitoylplasmenylethanolamine; 95, 2-aminobutyrate; 96, eicosapentaenoate (EPA 20:5n3); 97, dihomo-linolenate (20:3n3 or n6); 98, arachidonate (20:4n6); 99, docosahexaenoate (DHA 22:6n3); 100, 1-methylxanthine; 101,alpha-tocopherol acetate; 102, N-acetylmannosamine; 103, trans-4-hydroxyproline; 104, alpha-ketoglutarate; 105, cysteine; 106, isoleucylarginine; 107, valylarginine.
(DOCX)

S1 Table Gene expression primers for murine adenosine-metabolizing genes. (DOCX)

S2 Table Effect of diet and genetically-induced obesity on metrics of tumor formation by sex. P values are for Chi-square test (incidence) and for ANOVA (multiplicity, burden). N(females) = 5,8,7 and N(male) = 4,4,3 for Apc LF, HF and DbDb mice respectively.
(DOCX)
