# Supplementary material for: Diet- and Genetically-Induced Obesity Differentially Affect the Fecal Microbiome and Metabolome in Apc1638N Mice
Source: PLoS One. 2015 Aug 18;10(8):e0135758. doi: 10.1371/journal.pone.0135758 (PMC4540493; doi:10.1371/journal.pone.0135758)
Supplement: S1 Fig — Normalized adenosine concentration in fecal matter correlates with Il1b (A) and Tnf (B) but not Il4 (C) and Il6 (D) production in ex vivo colonic tissue. Relative abundance of Parabacteroides distasonis in fecal matter correlates with Il1b (E) but not Tnf (F), Il4 (G) and Il6 (H) production in ex vivo colonic tissue. (DOCX) [file pone.0135758.s001.docx]

**H.**

**G.**

**F.**

**E.**

**D.**

**C.**

**B.**

**A.**

p= 0.67

p= 0.11

R^2^= 0.23

p= 0.01

R^2^= 0.54

p= 1.3x10^-5^

p= 0.22

p= 0.27

p= 0.31

R^2^= 0.11

p= 0.05
