# Supplementary figures and images for: Diet- and Genetically-Induced Obesity Differentially Affect the Fecal Microbiome and Metabolome in Apc1638N Mice
Source: PLoS One. 2015 Aug 18;10(8):e0135758. doi: 10.1371/journal.pone.0135758 (PMC4540493; doi:10.1371/journal.pone.0135758)

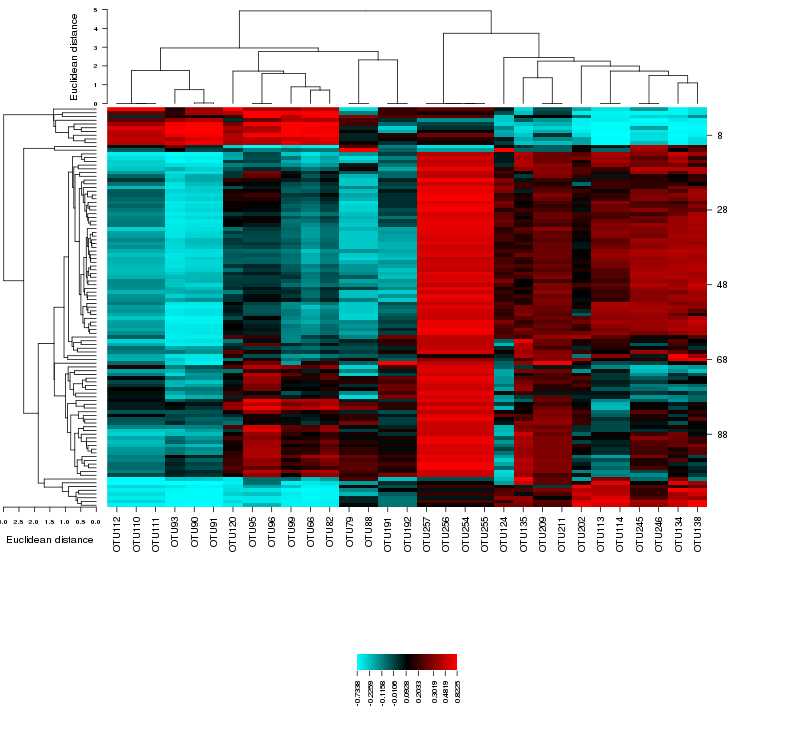

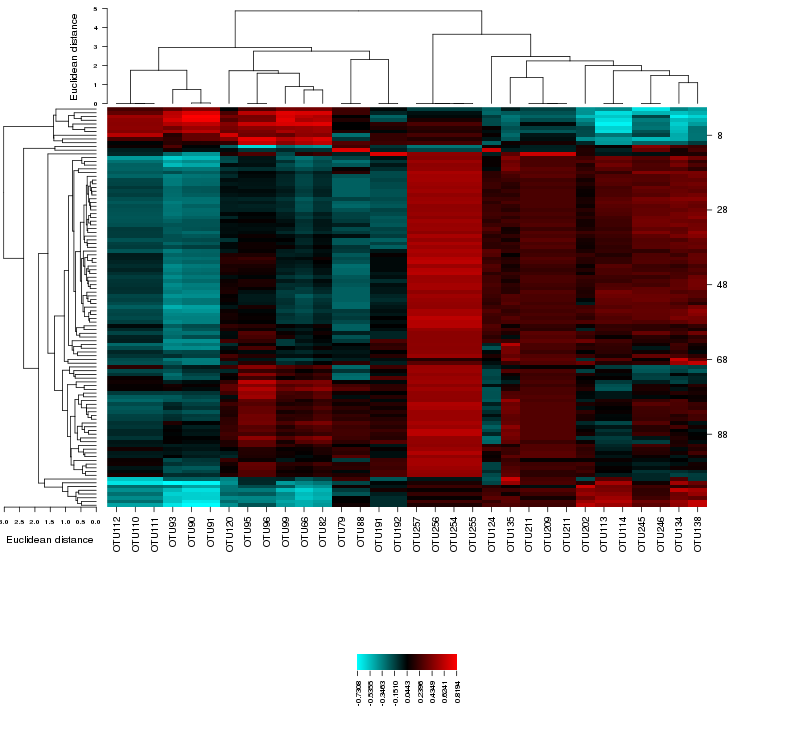


R value

Supplement: S2 Fig — Heat map consist of 107 metabolites significantly correlated with at least one OTU and 31 OTUs significantly correlated with at least 1 metabolite (q< 0.05). Color intensity indicates R value of association. For brevity phylum, class and lowest taxonomic classification of each OUT is listed (ie order, family and genus are omitted if OTU is a species). Metabolites by row: 1, 4-hydroxyphenylacetate; 2, betaine; 3, tyramine; 4, tyrosol; 5, lactate; 6, alpha-hydroxyisocaproate; 7, 2-hydroxy-3-methylvalerate; 8, N-acetylaspartate (NAA); 9, adenine; 10, N-acetylhistidine; 11, guanosine; 12, alpha-CEHC glucuronide; 13, 2-hydroxybutyrate (AHB); 14, lysylvaline; 15, 4-hydroxyphenylpyruvate; 16, lysylisoleucine; 17, glycylvaline; 18, tryptophylisoleucine; 19, serylphenyalanine; 20, alanylleucine; 21, alanylphenylalanine; 22, isoleucylleucine; 23, valylleucine; 24, valylphenylalanine; 25, threonylphenylalanine; 26, serylleucine; 27, isoleucylphenylalanine; 28, valyltyrosine; 29, tyrosylleucine; 30, asparagylleucine; 31, histidylleucine; 32, phenylalanylleucine; 33, isoleucyltyrosine; 34, histidyltyrosine; 35, tryptophylleucine; 36,seryltyrosine; 37, leucylleucine; 38, leucylphenylalanine; 39, val-val-val; 40,phenylalanylalanine; 41, leucylisoleucine; 42, phenylalanylvaline; 43, leucylthreonine; 44, leucylalanine; 45, valylisoleucine; 46, tyrosylvaline; 47, leucylmethionine; 48, methionylvaline; 49, threonylisoleucine; 50, leucylserine; 51, threonylvaline; 52, isoleucylisoleucine; 53, isoleucylvaline; 54, asparagylisoleucine; 55, phenylalanyltyrosine; 56, histidylisoleucine; 57, phenylalanylisoleucine; 58, tyrosylisoleucine; 59, leucylglycine; 60, valylvaline; 61, erucate (22:1n9); 62, 15-methylpalmitate (isobar with 2-methylpalmitate); 63, 2-oleoylglycerol (2-monoolein); 64, 1-stearoylglycerol (1-monostearin); 65, docosapentaenoate (n3 DPA 22:5n3); 66, alanine; 67, glucosylglycerol; 68, 3-hydroxypropanoate; 69, 1-palmitoylglycerophosphoglycerol; 70, uracil; 71, beta-alanine; 7 [file pone.0135758.s002.docx]
