# Supplementary material for: Diet- and Genetically-Induced Obesity Differentially Affect the Fecal Microbiome and Metabolome in Apc1638N Mice
Source: PLoS One. 2015 Aug 18;10(8):e0135758. doi: 10.1371/journal.pone.0135758 (PMC4540493; doi:10.1371/journal.pone.0135758)
Supplement: S1 Table — (DOCX) [file pone.0135758.s003.docx]

### Supplementary Table 1. Gene expression primers for murine adenosine-metabolizing genes.

| **Gene name** | **Gene symbol** | **mRNA Refseq accession#** | **Left primer** | **Right primer** |
| --- | --- | --- | --- | --- |
| adenosine deaminase | *Ada* | NM_007398.4 | GACACCCGCATTCAACAAAC | ATGCCTCTCTTCTTGCCAAA |
| adenosine kinase | *Adk* | NM_134079.4 | GAGAAGCACCTTGACCTGGA | TCAATACCGACTCTGGGGAG |
| S-adenosyl homocysteine hydrolase | *Ahcy* | NM_016661.3 | CGCCAGCATGTCTGATAAAC | CCTGGCATCTCATTCTCAGC |
| deoxycytidine kinase | *Dck* | NM_007832.4 | CTGGCTCCTTCATCGGACT | CCAGGCTTTCGTGTTTGTCT |
| ectonucleoside triphosphate diphosphohydrolase 1 | *Entpd1 (CD39)* | NM_009848.3 | AGCTGCCCCTTATGGAAGAT | GCCAAGATAGAGGTGAAACCA |
| ectonucleoside triphosphate diphosphohydrolase 3 | *Entpd3* | NM_178676.4 | CCTACTGCTTCTCAGCCCAC | CATGTAGCCAAGGGACCAGG |
| ectonucleoside triphosphate diphosphohydrolase 8 | *Entpd8* | NM_028093.1 | GTGTGCAGGTCAGAAGCAGA | CAGAGCCATGAAGACCCGTT |
| 5',3'-nucleotidase, cytosolic | *Nt5c* | NM_015807.1 | AGCAGTACGGAGCTCTGAGG | AGGGATGGGCTCCAAGTTTA |
| 5'-nucleotidase, cytosolic IA | *Nt5c1a* | NM_001085502.1 | ACGAGAATGAGCCCTTCAGC | AAGTCGCTTGTTCACAGCCT |
| 5'-nucleotidase, cytosolic IB | *Nt5c1b* | NM_027588.3 | GAACATCACGGAGCCCATCT | GCCTCTACCTTGATGGCAGT |
| 5'-nucleotidase, cytosolic II | *Nt5c2* | NM_029810.4 | TGACCGCTTACAGAATGCAG | TGGCTAAACTTCGGTTCACA |
| 5'-nucleotidase, cytosolic III | *Nt5c3* | NM_026004.3 | GAGAAAAACGGGCCGCAAG | TTGGCAGCGCCTCCTTTAAT |
| 5'-nucleotidase, cytosolic IIIB | *Nt5c3b* | NM_001102650.1 | GGTGGTTGGAGAGTCCACTG | TCCAGGATGTCACCAATGCC |
| 5' nucleotidase, ecto | *Nt5e (CD73)* | NM_011851.4 | CTTCATGAACATCCTGGGCT | AACGTTTCTGAGGAGGGGAT |
| 5’,3’-Nucleotidase, mitochondrial | *Nt5m* | NM_134029.2 | AGCCCCATCAAGATGTTCAA | TGGTCAACACAATCTGCTCC |
| purine-nucleoside phosphorylase | *Pnp* | NM_013632.4 | GGAAAGGGCAGGATTTCG | TTCAGTGTGTTGCAGAAGCC |
| purine-nucleoside phosphorylase 2 | *Pnp2* | NM_001123371.2 | AAGATTTGGGCGCCTCTGTC | CACTGCCACTTGAGGTCGAT |
| glyceraldehyde-3-phosphate dehydrogenase | *Gapdh* | NM_001289726.1 | TTGATGGCAACAATCTCCAC | CGTCCCGTAGACAAAATGGT |
