# Supplementary material for: Diet- and Genetically-Induced Obesity Differentially Affect the Fecal Microbiome and Metabolome in Apc1638N Mice
Source: PLoS One. 2015 Aug 18;10(8):e0135758. doi: 10.1371/journal.pone.0135758 (PMC4540493; doi:10.1371/journal.pone.0135758)
Supplement: S2 Table — P values are for Chi-square test (incidence) and for ANOVA (multiplicity, burden). N(females) = 5,8,7 and N(male) = 4,4,3 for Apc LF, HF and DbDb mice respectively. (DOCX) [file pone.0135758.s004.docx]

## Supplementary Table 2. Effect of diet and genetically-induced obesity on metrics of tumor formation by sex

| **Endpoint** | **Sex** | **LF** | | | **HF** | | | **DbDb** | | | **P** |
| --- | --- | --- | --- | --- | --- | --- | --- | --- | --- | --- | --- |
| Tumor Incidence | F | 0.20 | | | 0.63 | | | 1.00 | | | 0.016 |
|  | M | 0.50 | | | 0.75 | | | 1.00 | | | 0.34 |
|  | All | 0.33 | | | 0.67 | | | 1.00 | | | 0.008 |
| Tumors multiplicity (#/mouse) | F | 0.40 | ± | 0.40 | 1.00 | ± | 0.38 | 2.43 | ± | 0.72 | 0.050 |
|  | M | 0.50 | ± | 0.29 | 0.75 | ± | 0.25 | 2.00 | ± | 0.58 | 0.048 |
|  | All | 0.44 | ± | 0.24 | 0.92 | ± | 0.26 | 2.30 | ± | 0.52 | 0.004 |
| Total Tumor Burden (mm3) | F | 0.95 | ± | 0.95 | 13.20 | ± | 7.53 | 18.90 | ± | 63.08 | 0.217 |
|  | M | 1.53 | ± | 1.12 | 19.26 | ± | 6.86 | 6.28 | ± | 30.66 | 0.032 |
|  | All | 1.24 | ± | 0.70 | 15.22 | ± | 5.39 | 32.15 | ± | 11.25 | 0.030 |

P values are for Chi-square test (incidence) and for ANOVA (multiplicity, burden). N(females)= 5,8,7 and N(male)= 4,4,3 for Apc LF, HF and DbDb mice respectively.
